# Transcriptome analysis of lncRNA expression patterns in human congenital lung malformations

**DOI:** 10.1186/s12864-021-08204-x

**Published:** 2021-11-29

**Authors:** Weili Yang, Pu Zhao, Yun Liu, Ping Cao, Xiang Ji, Ya Gao, Peng Li, Jiwen Cheng

**Affiliations:** 1 Department of Pediatric Surgery, the Second Affiliated Hospital of Xi’an Jiaotong University, No. 157, Xiwu Road, Xi’an, 710004 Shaanxi China; 2 Department of Neonatology, the third Affiliated Hospital, Xi’an Jiaotong University, Xi’an, 710068 Shaanxi Province China; 3 Department of Respiratory Medicine, the Second Affiliated Hospital of Xi’an Jiaotong University, Xi’an, 710004 Shaanxi Province China

**Keywords:** Congenital lung malformations, Transcriptome analysis, lncRNA expression pattern, Co-expression network, Cis regulatory

## Abstract

**Objectives:**

To explore the long non-coding RNA (lncRNA) expression pattern of congenital lung malformations on a genome-wide scale and investigate their potential biological function in four subtypes of congenital lung malformations.

**Methods:**

We obtained both lesions and normal lung control tissues from the patients diagnosed with CPAM-I, CPAM-II, ILS, and ILS-CPAM, and underwent lobectomy (i.e., surgical removal of the whole lobe which contains the localized lesion as well as normal lung tissue). Then, we performed lncRNA transcriptome profiling in these tissues by RNA sequencing (RNA-seq). A comprehensive bioinformatics analysis was conducted to characterize the expression profiles and relevant biological functions and for multiple comparisons of lncRNA expression in the different subtypes of congenital lung malformation tissues. Furthermore, the lncRNA-mRNA co-expression network was constructed, and dysregulated mRNAs were functionally analyzed. Finally, gene set enrichment analysis (GSEA) was used to predict the potential molecular mechanism of the identified lncRNAs.

**Results:**

A total of 5921 lncRNA transcripts were identified between congenital lung malformations tissues and normal lung control tissues. Compared with normal lung control, 481of these expressed lncRNAs were upregulated and 142 were downregulated in CPAM-I, 91 were upregulated and 14 were downregulated in CPAM-II, 39 were upregulated and 38 were downregulated in ILS, and 201 were upregulated and 38 were downregulated in ILS-CPAM. Unsupervised clustering and principal component analysis of the expressed lncRNAs visualized the differences between normal lung control and different subtypes of congenital lung malformations samples. We also confirmed significant differences in the composition of differentially expressed genes (DEGs) and the differentially expressed lncRNAs (DE lncRNAs) between CPAM-I and other subtypes of congenital lung malformations, as well as in normal lung control tissues, and observed enrichment of DEGs in the regulation of the immune system, cell projection organization, and inflammatory pathways. Finally, we identified the lncRNA FLJ26850 might be related to congenital lung malformations via ZNF473.

**Conclusions:**

Significant differences in lncRNAs expression patterns were observed between different subtypes of congenital lung malformations and normal control. The lncRNA FLJ26850 might be related to congenital lung malformations via ZNF473.

**Supplementary Information:**

The online version contains supplementary material available at 10.1186/s12864-021-08204-x.

## Background

Congenital lung malformations include a wide spectrum of developmental abnormalities involving large airways, pulmonary parenchyma, and blood vessels; congenital pulmonary airway malformations (CPAM, previously known as congenital cystic adenomatoid malformations [CCAM]), pulmonary sequestration (further categorized into intralobar sequestrations [ILS] and extralobar sequestrations [ELS]), congenital lobar emphysema (CLE), and bronchogenic cysts are most commonly seen in clinics [1–3]. Typically, lesions occur sporadically without any family inheritance and are only occasionally accompanied by other deformities [4, 5]. Due to their rarity, few reliable data sources can be found on their overall prevalence. But the estimated prevalence of CPAM is 1 per 11,000 to 35,000 live births, comparable to congenital diaphragmatic hernia [4]. Given the increased risk of recurrent pneumonia and theoretical concern for malignant degeneration in later life, pediatric surgeons favor surgical removal of the affected lung in symptomatic infants [6].

Clinically, those lesions are simply grouped by cyst size (microcystic versus macrocystic) and origin of vascular supply (i.e., presence of an anomalous systemic arterial vessel [ILS or ELS], and pulmonary vs. systemic venous drainage) regardless of their heterogeneous and complex nature [3, 7]. CPAM is usually regarded as the most common type of fetal lung lesion, which was first classified into three subtypes in 1977, and later in 2002, it was reclassified into five subtypes by Stocker based on histology and the presumed site of origin during lung development, with CPAM-I speculatively originating from the tracheobronchial tree, through CPAM-VI, the latter thought to arise from distal alveolar regions [8–11]. Considering the presence of hybrid lesions (such as CPAM with an anomalous systemic arterial vessel, known as ILS combined with CPAM), it is a long-held suggestion that congenital lung abnormalities are part of a continuum of developmental abnormalities instead of separate entities, but the exact etiology and molecular pathogenesis of congenital lung lesions remain unknown [3, 12].

Found in every branch of life, long non-coding RNAs (lncRNAs) compose a category of long RNA transcripts with no protein-coding potentiality, enriched in the nucleus or cytoplasm [13]. According to the relationship with coding RNA transcripts, there are five groups of lncRNAs, including sense, anti-sense, intronic, intergenic, and divergent [14]. They can function as signals, sponges/decoys, scaffolds, and guides and play essential roles in various cellular processes, such as dosage compensation, imprinting, transcription/translation, alternative splicing, nuclear-cytoplasmic trafficking, and subcellular localization, etc. [15]. Thus, lncRNAs function critically in both transcriptional and post-transcriptional modifications [16].

To our best knowledge, no studies seem to investigate the regulatory roles of lncRNAs for congenital lung malformations on a genome-wide scale. In the present study, we conducted a combination of transcriptome analyses to explore the molecular pathogenesis of congenital lung malformations (specifically, CPAM, ILS, and hybrid lesions). Then, we determined the DE lncRNAs by using bioinformatics analysis and predicted the differentially expressed mRNAs. Subsequently, the interaction network among lncRNAs and mRNAs was constructed in patients with congenital lung malformations. This study may promote our understanding of the role of lncRNAs and associated pathways in human congenital lung malformations.

## Results

### Genome-wide profiling of the lncRNA expression associated with congenital lung malformations

**Fig. 1:**
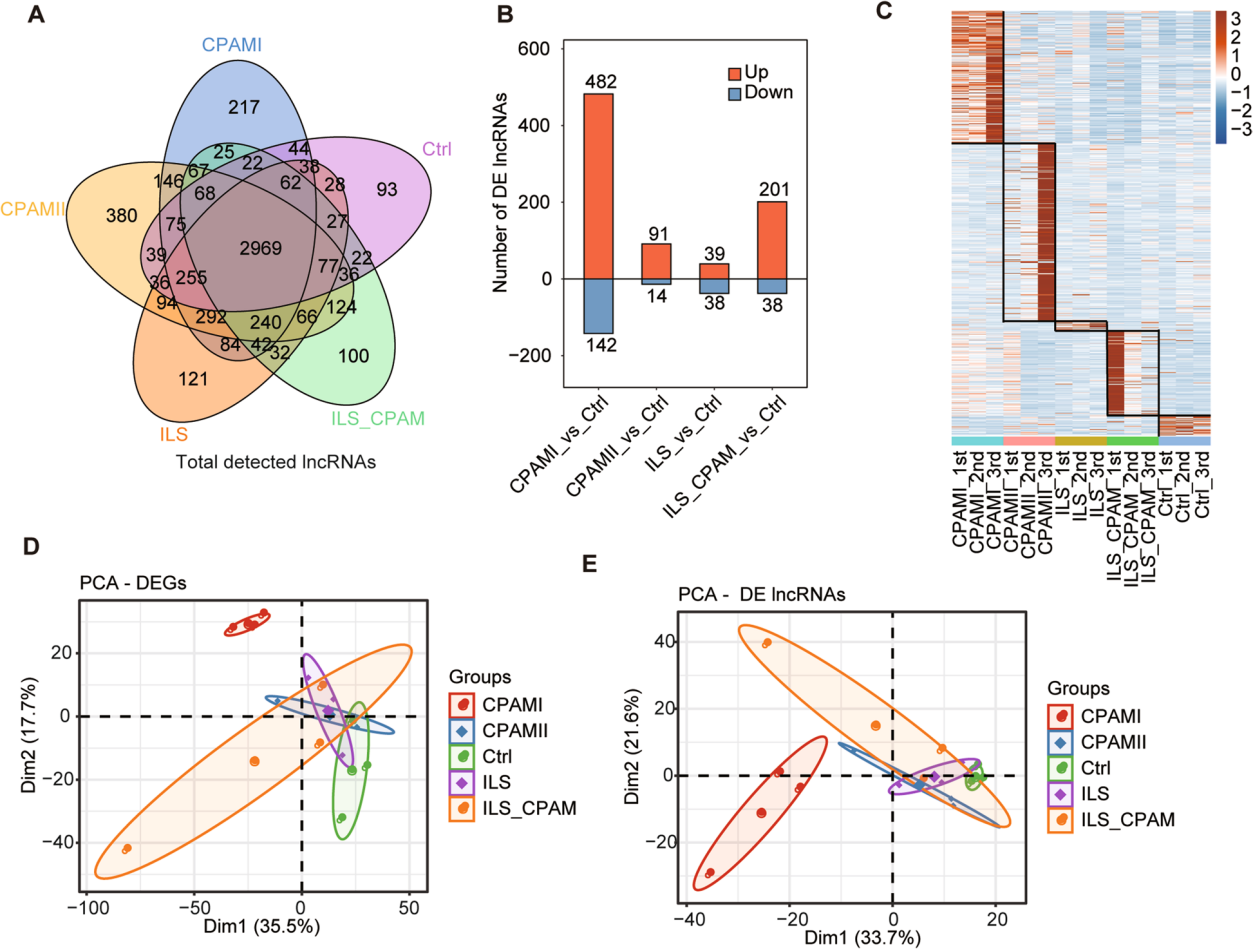
A comprehensive catalog of lncRNA expression pattern in congenital lung malformation tissues. **a.** The Venn diagram of detected lncRNA data from 15 samples (three CPAM-I, three CPAM-II, three ILS, three ILS hybrid CPAM, and three normal lung control samples obtained from patients undergoing lobectomy). **b.** The bar plot illustrates the number of differentially expressed genes between subtypes of congenital lung malformation with paired control lung tissue. **c.** Heatmap of all congenital lung malformation-specific lncRNAs. The color key from blue to red indicates the z-score color range. **d.** Principal component analysis (PCA) of 15 samples based on normalized differentiation gene expression level. All CPAM-I samples grouped together were used for the subsequent analyses. **e.** Principal component analysis (PCA) of 15 samples based on normalized DE lncRNA expression level

In the present study, we used a whole-genome RNA-seq dataset recently generated from 15 samples containing three CPAM-I, three CPAM-II, three ILS, three ILS-CPAM lung tissue samples, and three normal lung control samples from patients undergoing surgical resection of congenital lung malformations. A subset of lncRNAs showed remarkable expression changes in different subtypes of congenital lung malformations compared to the normal lung control tissues, as shown in the Venn diagram, the bar plot, and the clustered heatmaps (Fig. 1a-c).

Moreover, we observed that both the known lncRNAs and the novel lncRNAs showed remarkable expression changes in the different subtypes of congenital lung malformations tissues and normal lung control tissues using the Venn diagram (Fig. S1A and B). We also found gene length distribution and exon count of the known lncRNA, the novel lncRNA, and the protein-coding RNA were significantly different in the different subtypes of congenital lung malformations tissues and normal lung control tissues (Fig. S1C and D). Next, we observed the principal component of DEGs and DE lncRNAs in congenital lung malformations tissues and normal lung control tissues and found that significant differences in the composition of DEGs and lncRNAs between CPAM-I and other subtypes of congenital lung malformation, as well as in normal control (Fig. 1d and e, Fig. S1E and F). The above results indicated that significant differences in lncRNAs expression patterns existed among different subtypes of congenital lung malformations and normal control.

### Analysis of the lesion type specific-lncRNA and their co-expressed mRNAs in CPAM-I and other lesion tissues

**Fig. 2:**
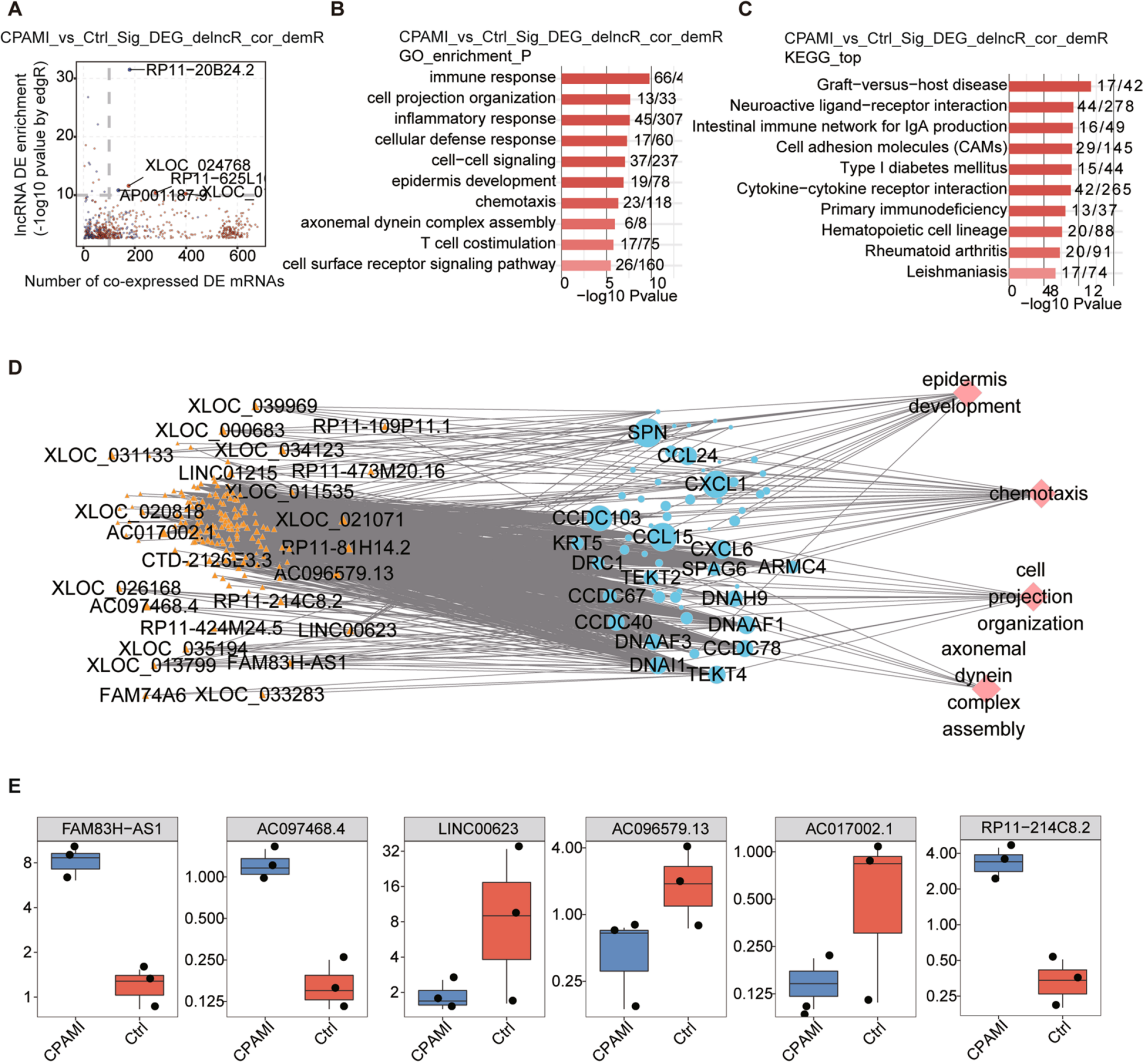
Co-expression network illustration of lncRNA-mRNA and functional analysis of target mRNAs in CPAM-I lung tissues. **a.** Scatter plot shows DE-lncRNAs of CPAM-I compared with control samples and the number of co-expressed DE mRNAs. Up and down-regulated lncRNAs are respectively labeled in red and blue points. Cutoffs of *p*-value < 0.01 and Pearson coefficient > 0.6 were applied to identify the co-expression pairs. **b.** Top 10 most enriched GO terms (biological process) by DE mRNAs co-expressed with DE-lncRNAs of CPAM-I lung tissues compared with control samples. **c.** The top 10 enriched KEGG pathways by DE mRNAs co-expressed with DE-lncRNAs of CPAM-I lung tissues compared with control samples. **d.** The co-expression network between the lncRNAs detected in the CPAM-I lung tissues and the co-expressed mRNAs involved in the four GO terms. lncRNA are plotted with yellow circles, co-expression mRNAs are plotted with light blue circles, mRNA-enriched GO terms are plotted with red circles. **e.** The expression level of the six lncRNAs in CPAM-I lung tissues

Given the distinction between CPAM-I and other subtypes of congenital lung malformations, we analyzed the DE lncRNAs and their co-expressed mRNAs in CPAM-I tissues. The scatter plot shows the DE lncRNAs of CPAM-I compared with normal lung control samples and the number of their co-expressed DEGs, and up and down-regulated lncRNAs (Fig. 2a, Fig. S2A-C). The GO analysis and KEGG pathway analysis of the co-expression mRNAs in CPAM-I and normal control tissues indicated enrichment in the immune response, cell projection organization, and inflammatory response; additionally, the co-expression mRNAs in CPAM-II and normal control tissues indicated enrichment in the immune response; the co-expression mRNAs in ILS and normal control tissues indicated enrichment in the cellular defense response, and co-expression mRNAs in ILS-CPAM and normal control tissues indicated enrichment in the immune response, cell projection organization, and cellular defense response (Fig. 2b and c, Fig. S2D-I). Moreover, we constructed the co-expression network between the lncRNAs detected in the CPAM-I lung tissues and co-expressed mRNAs and found four GO terms involved in this co-expression network (Fig. 2d). Subsequently, we detected the expression level of the top six most DE lncRNAs and found that lncRNA FAM83H-AS1, lncRNA AC097468.4, and lncRNA RP11-214C8.2 were significantly upregulated, and lncRNA LINC00623, lncRNA AC096579.13, and lncRNA AC017002.1 were significantly downregulated (Fig. 2e).

### WGCNA analysis of the lncRNA and mRNA co-expression in CPAM-I clinical samples

**Fig. 3:**
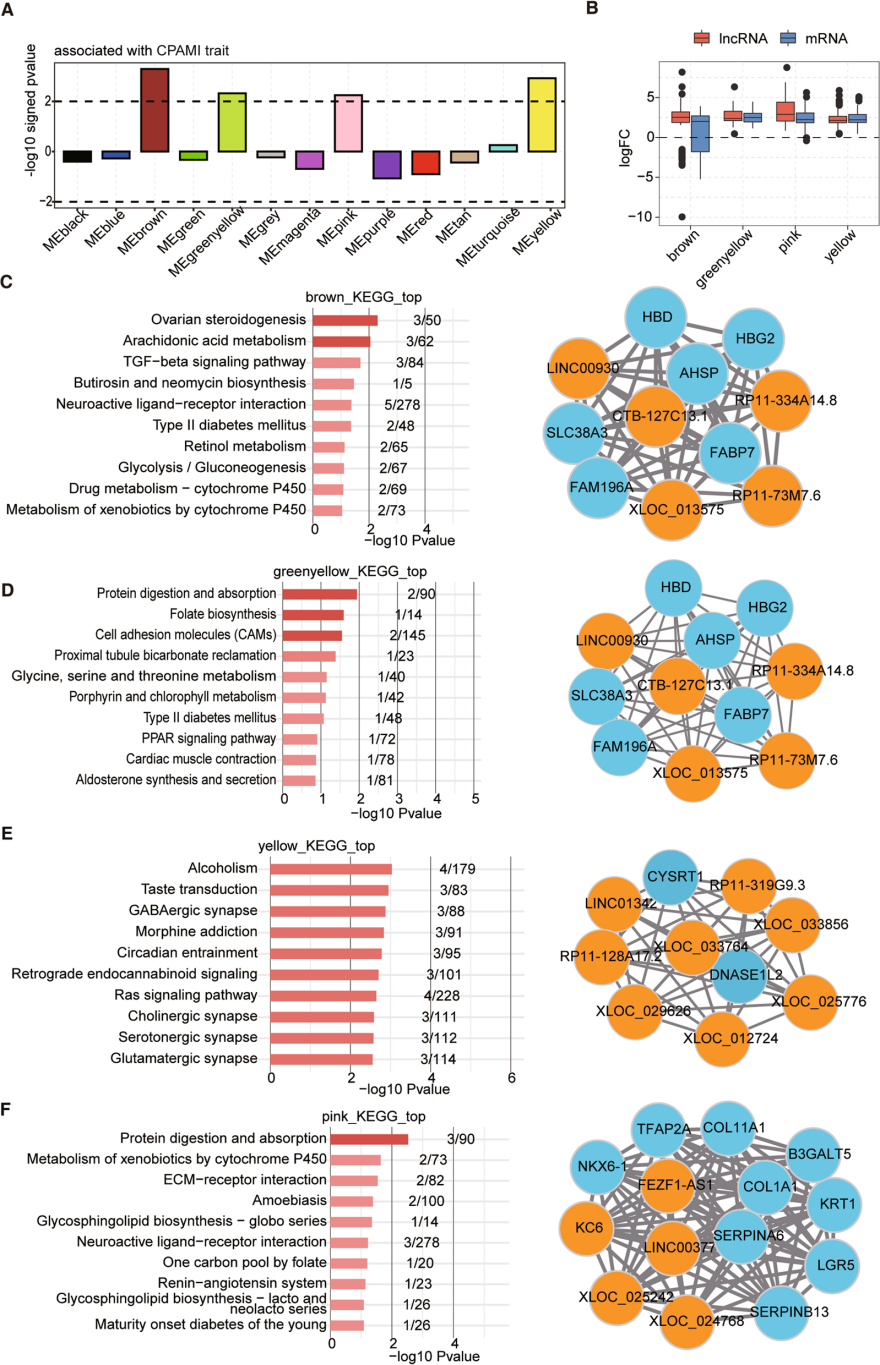
WGCNA analysis of expression pattern of differentially expressed lncRNAs and mRNAs in CPAM-I lung tissues. **a.** Signed association of module eigengenes with the diagnosis of CPAM-I. Positive values indicate modules with an increased expression in the disease samples. Negative values indicate modules with a decreased expression in the disease samples. The dashed lines signifies the disease-associated modules. **b.** The boxplot shows the expression fold change of mRNAs and lncRNAs from the four CPAM-I associated modules. c-f. The module plots display the top 10 hub genes and/or at least five top hub lncRNAs along with the GO terms enrichment of each of the four modules. Brown module (**c**), Greenyellow module (**d**), Yellow module (**e**), Pink module (**f**). Orange circles indicate lncRNAs and light blue circles indicate mRNAs

To further define the changes of DE lncRNAs in CPAM-I lung tissues, we evaluated the expression patterns of DE lncRNAs and co-expression mRNAs in CPAM-I lung tissues and normal control tissues by WGCNA analysis and found that CPAM-I samples had eight modules with a decreased expression, five modules with an increased expression in the disease samples with four modules at the location of the dashed line. These results represented the four modules involved in congenital lung malformations (Fig. 3a, Fig. S3A and B). Then, we detected the expression fold change of mRNAs and lncRNAs from the four CPAM-I associated modules and found that the fold changes in the expression levels of the corresponding mRNAs and lncRNAs were consistent (Fig. 3b). Furthermore, we analyzed the top 10 hub genes and/or at least five top hub lncRNAs along with the GO terms enrichment of each of the four modules. The brown module indicates the enrichment in the ovarian steroidogenesis and arachidonic acid metabolism, the green-yellow module indicates the enrichment in the protein digestion and absorption, folate biosynthesis, and cell adhesion molecules (CAMs), the yellow module indicates the enrichment in alcoholism, taste transduction, GABAergic synapse, and morphine addiction, and the pink module indicates the enrichment in the protein digestion and absorption (Fig. 3c-f).

### Analysis of cis-acting lncRNA-regulated mRNA network in congenital lung malformations

**Fig. 4:**
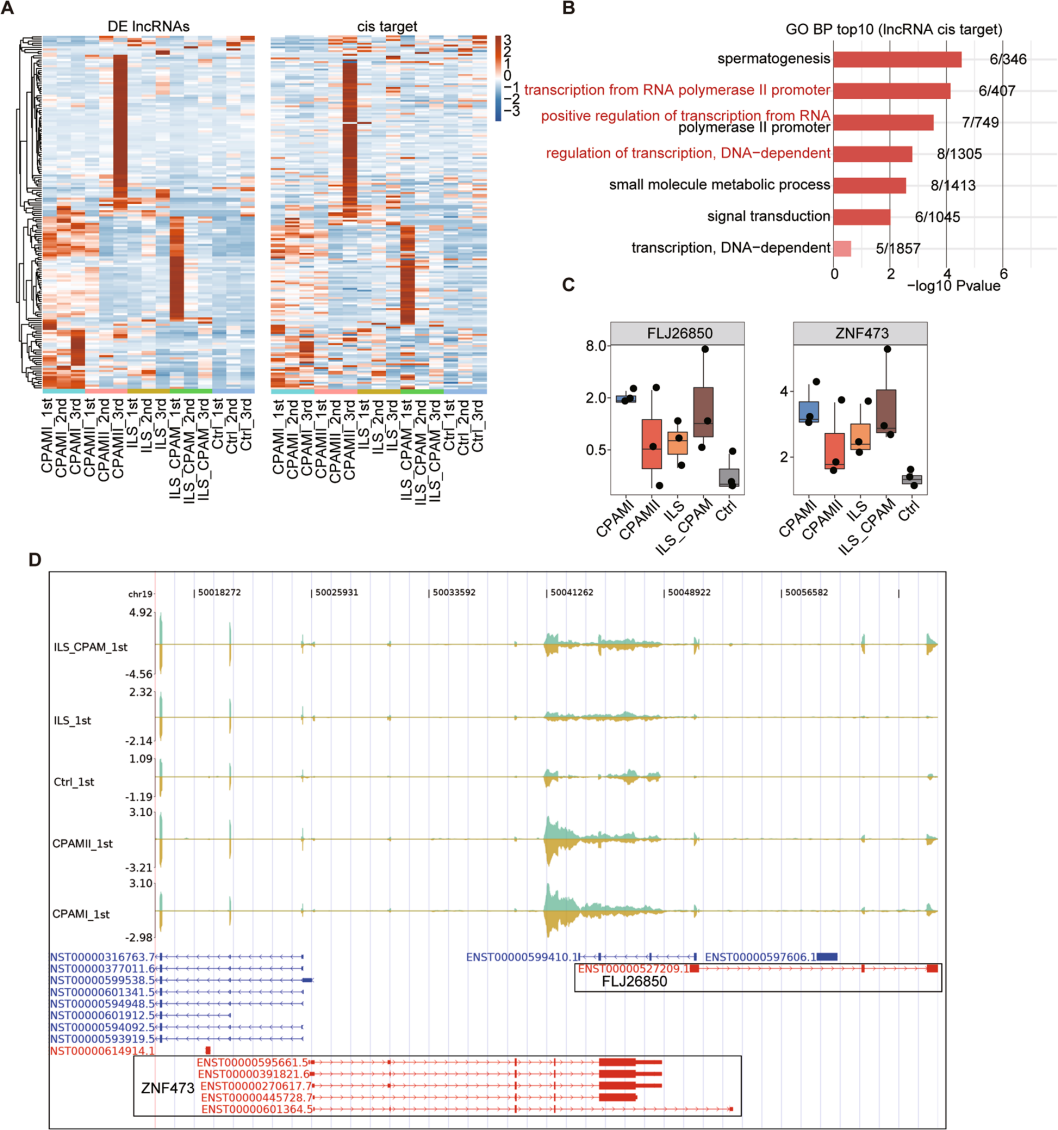
Cis-regulatory target genes of DE lncRNAs in all congenital lung malformation tissues. **a.** Heatmap shows the expression pattern of DE lncRNAs and their cis-regulatory genes. **b.** Top 10 most enriched GO terms of the cis-regulatory genes. **c.** The expression level of lncRNA FLJ26850 and its cis-regulatory target ZNF473 in all congenital lung malformation tissues. **d.** Visualization of lncRNA FLJ26850 and its cis-regulatory target ZNF473s

To characterize the function of DE lncRNAs in cis-regulation, their adjacent protein-encoding genes, which were placed 10 kb of lncRNAs downstream, were chosen to conduct co-expression analysis. In total, 151 lncRNA-mRNA pairs probably associated with cis-acting regulation were found (Fig. 4a). Cis-acting lncRNA-regulated mRNA indicated enrichment in the positive regulation of transcription from RNA polymerase-II promoter, regulation of transcription, and DNA dependent (Fig. 4b, Fig. S4A). Subsequently, we highlighted two DE lncRNAs FLJ26850 and PSORS1C3, both of which interacted with miRNA processing complexes and were typically upregulated in congenital lung malformations (Fig. 4c and d, Fig. S4B and C). These data showed that the dysregulation of lncRNAs, many of which are congenital malformation lung tissue-enriched, affected the mRNAs through the cis-regulatory target.

## Discussion

The first identification of congenital lung lesions was over a century, but their etiology and pathogenesis only have been partially unveiled [2, 7]. Different classification systems and terminologies have evolved to describe this disorder in clinical practice, yet the differential expression and regulatory mechanisms of lncRNAs in different subtypes of congenital lung malformations are unknown. In this study, we screened the genome-wide transcripts comprehensively to identify regulating lncRNAs in the different subtypes of congenital lung malformations with RNA-seq data. Our analysis suggested that the dysregulated lncRNAs were mostly involved in the following pathways: immune response, cell projection organization, and inflammation. Noticeably, the lncRNA FLJ26850 might be linked to congenital lung malformations via the ZNF473-mediated biological process. Overall, our results implicated the potential regulatory roles of several lncRNAs in congenital lung malformations.

The mammalian genome encodes a large number of lncRNAs, which play key functional roles in biological processes including cell proliferation and differentiation [17, 18]. In our study, many mRNAs were found regulated by multiple differential lncRNAs, such as HBD, HBG2, FAM196A, AHSP, and COL1A1. With the development of high-throughput technologies, large amounts of microarray and RNA- seq data have been obtained, but few studies have characterized lncRNA expression profile in congenital lung malformations. In our analysis, we characterized the expression profile of lncRNAs in congenital lung malformations by RNA-seq and identified 5921 expressed lncRNAs between different subtypes of congenital lung malformations tissues and normal lung control tissues. From the GO and pathway analyses of lncRNAs co-expressed mRNAs, we found that these significantly DE lncRNAs were associated with the immune response and inflammatory pathways. The results imply that they might play important roles in congenital lung malformations.

Congenital lung lesions usually display relatively mature airway structures of varying morphological features, indicating substantial heterogeneity in histology both within and across lesion types, even within individual lesions [19, 20]. Specifically, recent research has further reported unequal histopathologic characteristics and gene expression changes associated with the pathogenesis of these lesions [11, 21–23]. The gene expression dataset generated by our current study contributes to the comprehensive view of the transcriptional changes within various common types of congenital lung lesions. We observed a significant enrichment of known ZNF473 targets among the down-regulated transcripts. Also, a couple of molecular pathways are suspected to involve in the pathogenesis by the GSEA data, which may provide certain directions for future studies.

## Conclusion

In this study, we ascertained the differential expression of lncRNAs in congenital lung malformations tissues compared with normal lung control tissues using RNA-seq. And the results of the GO and pathway analyses showed that certain lncRNAs might play key roles in the development of congenital lung malformations. Moreover, in these lncRNAs, we found the most dysregulated lncRNA FLJ26850 in congenital lung malformations. It could be used as a prognostic biomarker and has the potential to be a diagnostic biomarker and a new therapeutic target for congenital lung malformations. Although our findings are preliminary, while our study promotes understanding of the pathogenesis of congenital lung malformations and provides potential biomarkers for diagnosis and treatment of congenital lung malformations.

### Materials and methods

All methods were carried out in accordance with relevant guidelines and regulations.

### Patients and specimens

We recruited 1-year-old infants with a confirmed diagnosis of CPAM-I (*N* = 3, mean ± SE: 2.89 ± 0.67 months, including one male), CPAM-II (*N* = 3, mean ± SE: 3.15 ± 0.84 months, including one male), ILS (*N* = 3, mean ± SE: 6.37 ± 1.96 months, including two males) and ILS- CPAM (*N* = 3, mean ± SE: 4.03 ± 0.97 months, including two males) into our study. Normal lung control samples (*N* = 3, mean ± SE:4.81 ± 0.87 months, includes 2 males) were obtained from patients undergoing resection of congenital lung malformations at the Second Affiliated Hospital of Xi’an Jiaotong University from May 2017 to August 2018. Specimens from each patient were then divided into two portions. The first portion was fixed in buffered formalin for histopathologic examination, and the second one was immediately snap-frozen in liquid nitrogen for RNA-seq analysis. The study protocol was approved by the Institutional Ethics Board of Xi’an Jiaotong University and written informed consent was obtained from the guardians of all subjects.

### RNA preparation and sequencing

Total RNA including the lncRNA (miRNA) fraction was isolated with High Pure miRNA isolation kit (Cat no: 05080576001, Roche, Penzberg, Germany) using the one-column protocol according to the manufacturer’s recommendation. RNA quantity was measured on a Qubit fluorometer with the Qubit RNA Assay Kit (Life Technologies, Eugene, OR, USA) and also on the NanoDrop-1000 instrument (Thermo Fisher Scientific Inc., Waltham, USA) to determine the purity values (OD260/280, OD260/230). RNA quality analysis was performed on an Agilent Bioanalyzer microcapillary electrophoresis system with the RNA 6000 Pico Kit (Agilent, Santa Clara, CA, USA).

### RNA-Seq raw data clean and alignment

We first discarded raw reads with more than 2-N bases. Then, we used FASTX-Toolkit (Version 0.0.13) to trim adaptors and low-quality bases from raw sequencing reads. We also dropped reads shorter than 16 nt. Next, we aligned those clean reads to the GRCH38, version 23 (Ensembl 81) (https://www.ebi.ac.uk/about/news/service-news/ensembl-version-81-release) using TopHat v2 with four mismatches. The RNAseq mapping statistics can be found in Supplementary Material Table 1). Further, we used uniquely mapped reads to count the number of gene reads and calculate FPKM (fragments per kilobase of transcript per million fragments mapped). The cleaned data for each sample were saved in FASTQ format. FastQC (http://www.bioinformatics.babraham.ac.uk/projects/fastqc/) was used to assess the quality of the raw reads.

### Reverse transcription-quantitative polymerase chain reaction (RT-qPCR) [24]

After RNA isolation, M-MLV reverse transcriptase (Invitrogen, USA) was used for synthesizing cDNA according to the manufacturer’s instructions. Subsequently, we performed RT-qPCR by SYBR Green assays in a total reaction volume of 20 μL, including 0.8 μL of PCR Forward Primer (10 μM), 0.8 μL of PCR Reverse Primer (10 μM), 2 μL of cDNA, 5 μL of 2 × Master Mix and 12.8 μL of double distilled water. The RT-qPCR cycling conditions consisted of 95 °C for 3 min; then 35 cycle amplification for 35 s at 95 °C, 45 s at 58 °C, 30 s at 72 °C; followed by 10 min at 72 °C. β-actin was used as a housekeeping gene for normalization, and the relative change in gene expression was analyzed by the 2^−ΔΔCT^ method.

### Principal component analysis, hierarchical clustering and visualization

Firstly, we used the TCGAanalyze_Normalization function for quantile-normalization of raw counts both within and across samples; then, to control heteroscedasticity, the counts were transformed using the varianceStabilizingTransformation function in DESeq2 (chosen over log transformation to minimize variance at low count values) [25]. We used the prcomp function in R to conduct principal component analysis (PCA)(https://cloud.r-project.org/package=factoextra) [26]. To get hierarchical clusters, the Euclidean distance was used to measure the dissimilarity following the Ward2 criterion [27]. To determine how much variance may be explained by the variables, we first used the lm function to construct multiple linear regression models for response variables “PC1” and “Cluster”, respectively. Next, the relaimpo package was chosen to determine the relative contribution of explanatory variables to the variance [28]. The PCA results were plotted using the plotPCA function in DESeq2 for visualization [25]. All heatmaps were constructed with the ComplexHeatmap R package [29].

### Correlation and co-expression network analysis

After calculating the Pearson correlation coefficient between lncRNAs and mRNAs based on differentially expressed mRNAs and lncRNAs, those lncRNAs and mRNAs whose expression levels showed meaningful correlations (*P-value* < 0.05) were subjected to the co-expression analysis. For each gene pair (including lncRNAs and protein-coding genes), we used the WGCNA package in R to calculate the Pearson correlation coefficients and corresponding *P*-values [30]. All *P*-values were adjusted via Bonferroni correction using the multtest R package to account for multiple testing [31]. Gene/lncRNA clusters with high interconnections were detected by Markov clustering (MCL) [32]. Bonferroni-adjusted *P*-values (cutoff: 0.01) were used as edge weights for MCL.

### Gene Ontology (GO) and pathway enrichment analysis

We performed Gene Ontology analysis (GO, http://geneontology.org/) for the meaningful annotations of genes and gene products, which has covered various domains of biological processes, cellular components, and molecular functions. The -log10 (*P*-value) denotes enrichment score, which represents the significance of GO term enrichment among differentially expressed genes. We also conducted Kyoto Encyclopedia of Genes and Genomes pathway analysis (KEGG, http://www.kegg.jp/) [33] to harvest pathway clusters along with our knowledge on the molecular interactions and reaction networks in differentially regulated gene profiling. Furthermore, the -log10 (*P*-value) denotes enrichment score showing the significance of the pathway correlations.

### Cis-regulation prediction [34]

A cis-regulator exerts its function on a neighboring gene located at the same chromosome [35]. After we identified the significantly changed lncRNAs whose expression levels were correlated with that of mRNAs, they were subjected to cis prediction. The genomic localization of the paired lncRNAs and mRNAs was identified. If the nearby gene is less than 10 kb downstream away from the lncRNA, it is assumed to be the putative target regulated by that lncRNA in a cis manner. With this criterion, we enriched those mRNAs co-expressed with lncRNAs significantly overlapped with the target genes to construct the lncRNAs-mRNAs network.

## Supplementary Information

**Additional file 1: Supplementary Material Table 1.** RNA-seq Mapping Statistics.

**Additional file 2: Figure S1.** Characteristics of lncRNAs detected in all congenital lung malformation tissues. A. Venn diagram of detected known lncRNA in human congenital lung malformation tissues.s At least two samples with RPKM> = 0.2 were considered to be detected in the group. B. Venn diagram of detected novel lncRNA (right) in human congenital lung malformation tissues. At least two samples with RPKM> = 0.2 were considered to be detected in the group. C. Distribution of gene length distribution of known lncRNA, novel lncRNA, and protein coding RNA. The length density distribution was generated by density function in R. D. Distribution of exon count of known lncRNA, novel lncRNA, and protein-coding RNA. E-F. Principal component analysis (PCA) of all samples based on all normalized mRNAs (E) and lncRNAs (F) expression levels. The samples were grouped by disease state and the ellipse for each group is the confidence ellipse.

**Additional file 3: Figure S2.** Co-expression network illustration between DElncRNAs and DEmRNAs. A-C. Scatter plots show DE lncRNAs by CCAM II (A), ILS_CCAM (B), ILS (C) compared with control samples and their number of co-expressed DE mRNAs. Red points denote up-regulated lncRNAs involved in co-expression pairs, and blue points denote down-regulated lncRNAs. Cutoffs of *P*-value < 0.05 and Pearson coefficient > 0.6 were applied to identify the co-expression pairs. D. The top 10 most enriched GO terms (molecular process) by DE mRNAs co-expressed with DE-lncRNAs of CCAM II lung tissues compared with control samples. E. The top 10 enriched KEGG pathways by DE mRNAs co-expressed with DE-lncRNAs of CCAM II lung tissues compared with control samples. F. The top 10 most enriched GO terms (molecular process) by DE mRNAs co-expressed with DE-lncRNAs of ILS lung tissues compared with control samples. G. The top 10 enriched KEGG pathways by DE mRNAs co-expressed with DE-lncRNAs of ILS lung tissues compared with control samples. H. The top 10 most enriched GO terms (molecular process) by DE mRNAs co-expressed with DE-lncRNAs of ILS_CCAM lung tissues compared with control samples. I The top 10 enriched KEGG pathways by DE mRNAs co-expressed with DE-lncRNAs of ILS_CCAM lung tissues compared with control samples.

**Additional file 4: Figure S3.** WGCNA analysis of all expressed lncRNAs and mRNAs. A. Hierarchical cluster dendrogram of all differentially expressed lncRNAs modules. Modules corresponding to branches are labeled with colors indicated by the color bands underneath the tree. B. Module-trait associations as computed by an LME model with all factors on the x-axis used as covariates. All Pearson’s correlation values and *p* values are displayed.

**Additional file 5: Figure S4.** Cis regulatory genes of DE lncRNAs. A. Top 10 most enriched Reactome pathways of cis-regulatory genes. B. Expression level of lncRNA PSORS1C3 and its cis-regulatory target POU5F1. C. Visualization of lncRNA PSORS1C3 and its cis-regulatory target POU5F1.

## Data Availability

All the raw data has been deposited to the GEO (Gene Expression Omnibus), with the accession number of GSE179404 (https://www.ncbi.nlm.nih.gov/geo/query/acc.cgi?acc=GSE179404).

## Abbreviations

lncRNA: Long non-coding RNA
DEGs: Differentially expressed genes
DE lncRNAs: Differentially expressed lncRNAs
CPAM: Congenital pulmonary airway malformations
ILS: Intralobar sequestrations
ELS: Extralobar sequestrations
CLE: Congenital lobar emphysema
RT-qPCR: Reverse transcription-quantitative polymerase chain reaction
PCA: Principal component analysis
GO: Gene ontology
RNA-seq: RNA sequencing
CAMs: Cell adhesion molecules
