# Supplementary material for: Transcriptome analysis of lncRNA expression patterns in human congenital lung malformations
Source: BMC Genomics. 2021 Nov 29;22:861. doi: 10.1186/s12864-021-08204-x (PMC8630864; doi:10.1186/s12864-021-08204-x)
Supplement: Supplementary file 1 — Additional file 1: Supplementary Material Table 1. RNA-seq Mapping Statistics. [file 12864_2021_8204_MOESM1_ESM.docx]

Supplementary Material Table 1. RNA-seq Mapping Statistics

| SampleID | Raw Reads | Clean Reads | Clean Percentage | Raw base | Clean base | Base Percentage | Unique Tag | Q20 | Q30 | GC Percentage | DUP |
| --- | --- | --- | --- | --- | --- | --- | --- | --- | --- | --- | --- |
| CPAMI_1st | 84069428 | 80872144 | 96.20% | 12.61G | 11.76G | 93.25% | 45539969(56.31%) | 97.44% | 92.81% | 50.00% | 67.48% |
| CPAMI_2nd | 84695326 | 81994882 | 96.81% | 12.70G | 11.91G | 93.75% | 45194716(55.12%) | 97.70% | 93.43% | 51.00% | 69.23% |
| CPAMI_3rd | 83431214 | 80230804 | 96.16% | 12.51G | 11.65G | 93.07% | 37666200(46.95%) | 97.43% | 92.81% | 51.00% | 78.22% |
| CPAMII_1st | 77694168 | 74661177 | 96.10% | 11.65G | 10.85G | 93.09% | 36260436(48.57%) | 97.52% | 92.99% | 51.00% | 74.61% |
| CPAMII_2nd | 95367314 | 92238017 | 96.72% | 14.31G | 13.40G | 93.65% | 50724414(54.99%) | 97.62% | 93.25% | 50.00% | 68.68% |
| CPAMII_3rd | 78825214 | 77444666 | 98.25% | 11.82G | 11.17G | 94.47% | 43489377(56.16%) | 98.17% | 95.03% | 52.00% | 64.60% |
| ILS_1st | 90164210 | 86796548 | 96.26% | 13.52G | 12.62G | 93.32% | 50223240(57.86%) | 97.45% | 92.84% | 50.00% | 66.09% |
| ILS_2nd | 86224798 | 83227559 | 96.52% | 12.93G | 12.10G | 93.59% | 46839171(56.28%) | 97.55% | 93.06% | 51.00% | 67.42% |
| ILS_3rd | 63726522 | 61568712 | 96.61% | 9.56G | 8.96G | 93.68% | 31004581(50.36%) | 97.62% | 93.16% | 50.00% | 73.08% |
| ILS_CPAM_1st | 146693396 | 139927528 | 95.39% | 22.00G | 20.25G | 92.02% | 71355427(50.99%) | 97.38% | 92.91% | 49.00% | 75.44% |
| ILS_CPAM_2nd | 94956204 | 91936045 | 96.82% | 14.24G | 13.37G | 93.89% | 45646741(49.65%) | 97.65% | 93.31% | 49.00% | 73.34% |
| ILS_CPAM_3rd | 85438088 | 82592797 | 96.67% | 12.82G | 12.01G | 93.68% | 47927422(58.03%) | 97.62% | 93.25% | 50.00% | 65.99% |
| Control_1st | 105294618 | 99459006 | 94.46% | 15.79G | 14.40G | 91.19% | 44398973(44.64%) | 97.16% | 92.37% | 50.00% | 82.43% |
| Control_2nd | 78600310 | 75727221 | 96.34% | 11.79G | 11.01G | 93.41% | 43947887(58.03%) | 97.48% | 92.88% | 50.00% | 65.59% |
| Control_3rd | 70561884 | 69422924 | 98.39% | 10.58G | 10.02G | 94.70% | 34296815(49.40%) | 98.27% | 95.25% | 50.50% | 70.58% |
