# Supplementary figures and images for: Transcriptome analysis of lncRNA expression patterns in human congenital lung malformations
Source: BMC Genomics. 2021 Nov 29;22:861. doi: 10.1186/s12864-021-08204-x (PMC8630864; doi:10.1186/s12864-021-08204-x)

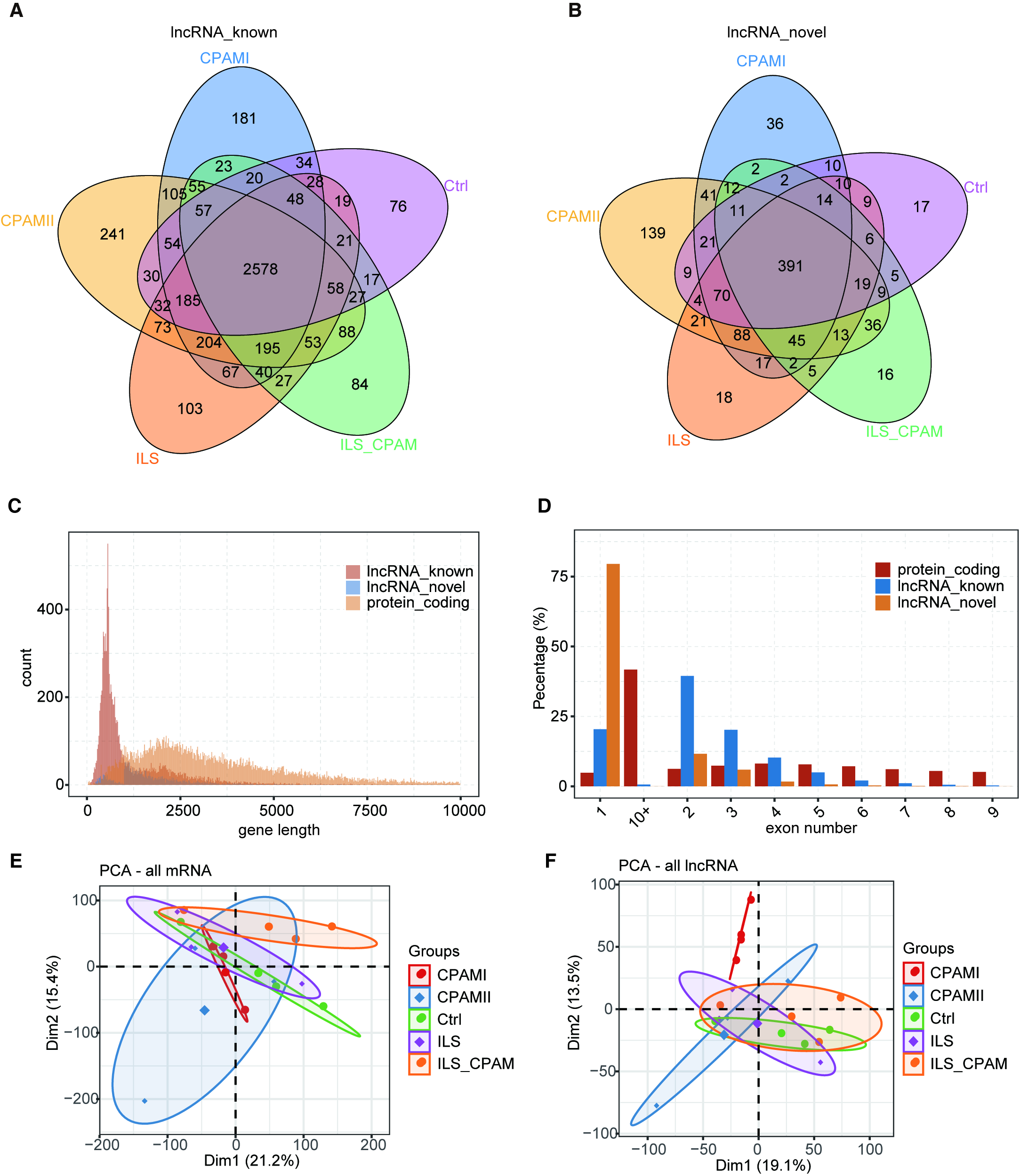

Supplement: Supplementary file 2 — Additional file 2: Figure S1. Characteristics of lncRNAs detected in all congenital lung malformation tissues. A. Venn diagram of detected known lncRNA in human congenital lung malformation tissues.s At least two samples with RPKM> = 0.2 were considered to be detected in the group. B. Venn diagram of detected novel lncRNA (right) in human congenital lung malformation tissues. At least two samples with RPKM> = 0.2 were considered to be detected in the group. C. Distribution of gene length distribution of known lncRNA, novel lncRNA, and protein coding RNA. The length density distribution was generated by density function in R. D. Distribution of exon count of known lncRNA, novel lncRNA, and protein-coding RNA. E-F. Principal component analysis (PCA) of all samples based on all normalized mRNAs (E) and lncRNAs (F) expression levels. The samples were grouped by disease state and the ellipse for each group is the confidence ellipse. [file 12864_2021_8204_MOESM2_ESM.tif]

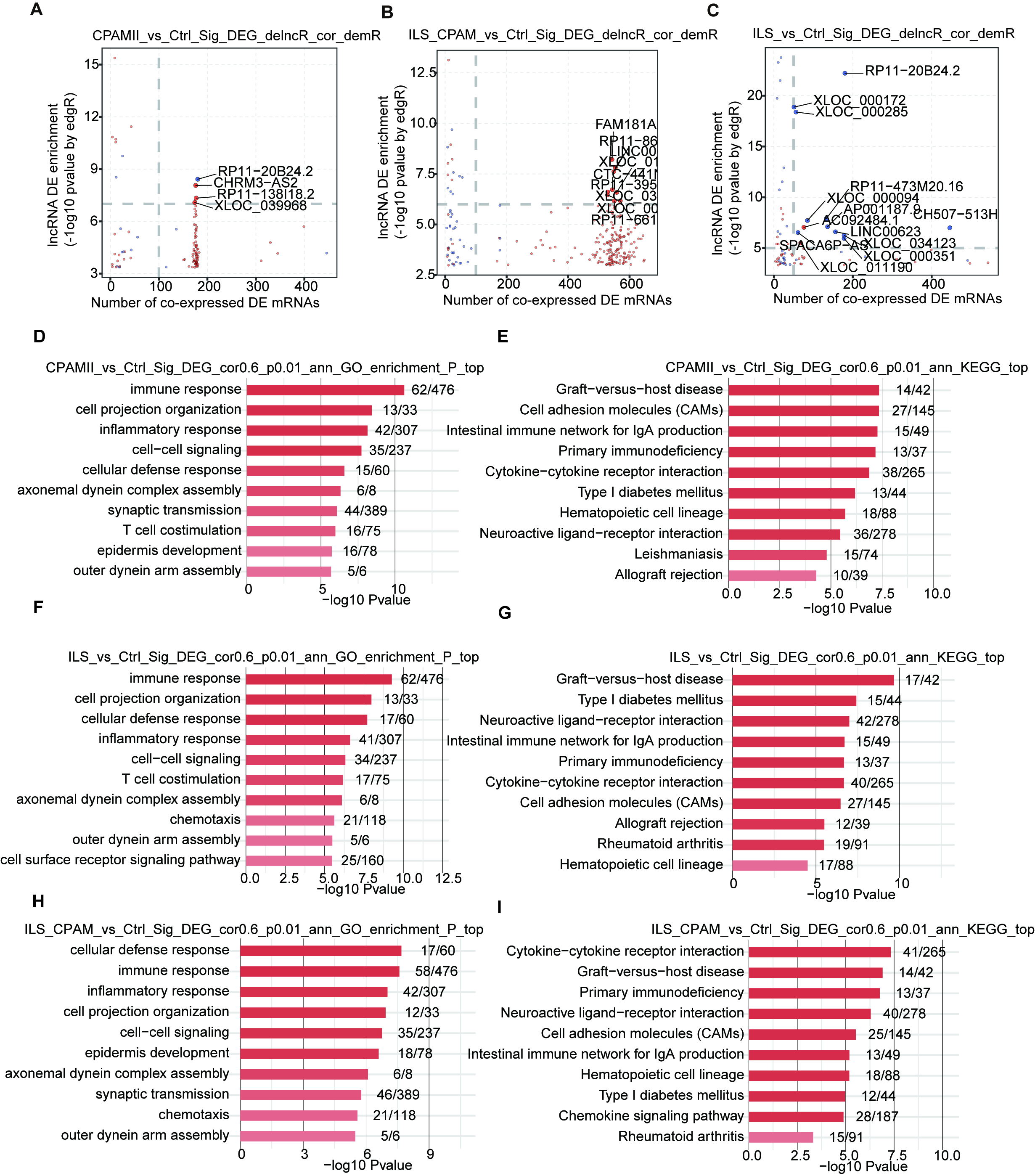

Supplement: Supplementary file 3 — Additional file 3: Figure S2. Co-expression network illustration between DElncRNAs and DEmRNAs. A-C. Scatter plots show DE lncRNAs by CCAM II (A), ILS_CCAM (B), ILS (C) compared with control samples and their number of co-expressed DE mRNAs. Red points denote up-regulated lncRNAs involved in co-expression pairs, and blue points denote down-regulated lncRNAs. Cutoffs of P-value < 0.05 and Pearson coefficient > 0.6 were applied to identify the co-expression pairs. D. The top 10 most enriched GO terms (molecular process) by DE mRNAs co-expressed with DE-lncRNAs of CCAM II lung tissues compared with control samples. E. The top 10 enriched KEGG pathways by DE mRNAs co-expressed with DE-lncRNAs of CCAM II lung tissues compared with control samples. F. The top 10 most enriched GO terms (molecular process) by DE mRNAs co-expressed with DE-lncRNAs of ILS lung tissues compared with control samples. G. The top 10 enriched KEGG pathways by DE mRNAs co-expressed with DE-lncRNAs of ILS lung tissues compared with control samples. H. The top 10 most enriched GO terms (molecular process) by DE mRNAs co-expressed with DE-lncRNAs of ILS_CCAM lung tissues compared with control samples. I The top 10 enriched KEGG pathways by DE mRNAs co-expressed with DE-lncRNAs of ILS_CCAM lung tissues compared with control samples. [file 12864_2021_8204_MOESM3_ESM.tif]

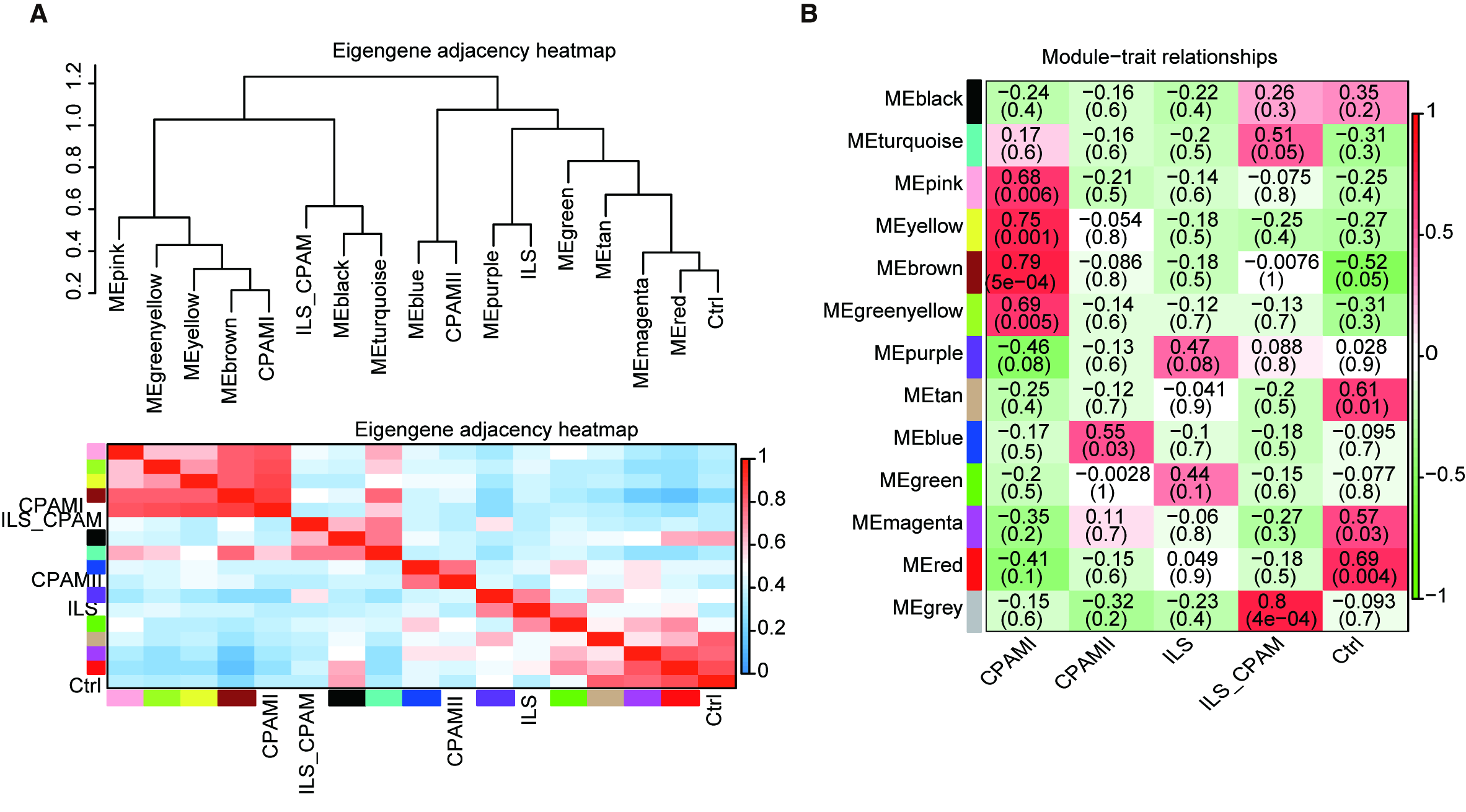

Supplement: Supplementary file 4 — Additional file 4: Figure S3. WGCNA analysis of all expressed lncRNAs and mRNAs. A. Hierarchical cluster dendrogram of all differentially expressed lncRNAs modules. Modules corresponding to branches are labeled with colors indicated by the color bands underneath the tree. B. Module-trait associations as computed by an LME model with all factors on the x-axis used as covariates. All Pearson’s correlation values and p values are displayed. [file 12864_2021_8204_MOESM4_ESM.tif]

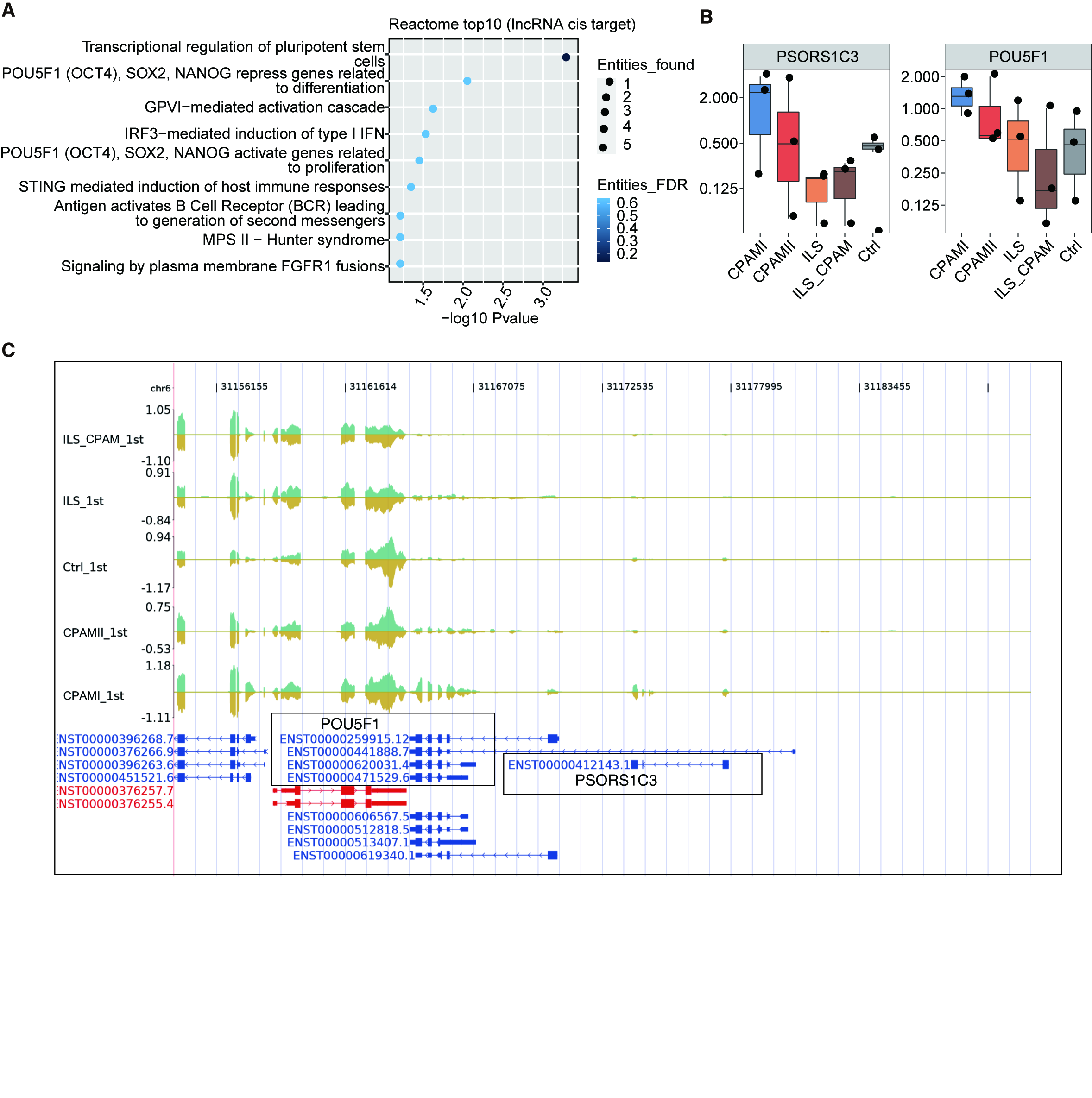

Supplement: Supplementary file 5 — Additional file 5: Figure S4. Cis regulatory genes of DE lncRNAs. A. Top 10 most enriched Reactome pathways of cis-regulatory genes. B. Expression level of lncRNA PSORS1C3 and its cis-regulatory target POU5F1. C. Visualization of lncRNA PSORS1C3 and its cis-regulatory target POU5F1. [file 12864_2021_8204_MOESM5_ESM.tif]
